# Endorsement of scientific inquiry promotes better evaluation of climate policy evidence

**DOI:** 10.1007/s10584-023-03535-y

**Published:** 2023-05-19

**Authors:** Jessica E. Hughes, James D. Sauer, Aaron Drummond, Laura E. Brumby, Matthew A. Palmer

**Affiliations:** 1 Present Address: School of Psychological Sciences, University of Tasmania, Tasmania, Australia; 2 School of Psychology, Massey University, Palmerston North, New Zealand

**Keywords:** Decision-making, Attitudes towards science, Motivated reasoning, Climate change policy, Deficit model, Scientific literacy, Scientific inquiry

## Abstract

Public and scientific consensus about climate change do not align. Problematically, higher scientific knowledge has been associated with lower acceptance of climate information among those with more conservative socio-political ideologies. Positive attitudes towards science can attenuate this effect. We investigated the association between *endorsement of scientific inquiry* (ESI) and decision-making with scientific evidence about climate policies. Participants rated support for 16 climate policies accompanied by weaker or stronger evidence. In study 1 (*N* = 503), higher ESI was associated with greater discernment between strongly and weakly evidenced climate policies, irrespective of worldview. In studies 2 (*N* = 402) and 3 (*N* = 600), an ESI intervention improved discrimination, and, in study 3, increased ESI specifically for hierarchical/individualistic participants. Unlike ESI, the link between scientific knowledge and evaluation of evidence was influenced by worldview. Increasing ESI might improve the evaluation of scientific evidence and increase public support for evidence-based climate policies.

**Supplementary Information:**

The online version contains supplementary material available at 10.1007/s10584-023-03535-y.

## Introduction

‘It is now unequivocal that human influence is causing climate change, making extreme events more frequent and more severe…Global warming of 1.5 and 2.0 degrees Celsius will be exceeded during this century unless immediate, rapid, and large-scale reductions in greenhouse gas emissions, especially of carbon dioxide and methane, occur in the nearest future’ (Lee 2021).

Climate change threatens the global population (International Panel on Climate Change [IPCC] 2018; World Health Organisation 2018). Although over 97% of climate scientists agree human factors cause climate change (Cook et al. 2016), public agreement has been reported between 47 and 62% (Leiserowitz et al. 2018; Leviston et al. 2013; Milfont et al. 2017). Evidence-based policies can mitigate climate change if they receive public support (A. Drummond et al. 2016, 2018; Leiserowitz 2019; McCright et al. 2013; Miller 2010). This support often depends upon public acceptance of the underlying scientific evidence: coordinated action is needed urgently to prevent catastrophic climate change. We investigated how attitudes towards science affect the evaluation of scientific evidence and support for evidence-based climate policies.

Public decision-making is not entirely rational (Cook and Lewandowsky 2016; Druckman and McGrath 2019) and, for controversial topics, motivated reasoning may lead individuals to prioritise opinions or norms of socio-political in-groups over scientific evidence (DiMaggio 1997; Druckman and McGrath 2019; Hornsey 2021; Kahan and Braman 2006; Kahan et al. 2007; Kunda 1990). Climate change is one area where attitudes on the socio-political right tend to be less consistent with the scientific consensus compared to the socio-political left (e.g. Cook and Lewandowsky 2016). Cultural cognition theory suggests this may occur due to differences in risk appraisal. According to this theory, individuals with stronger hierarchical/individualistic worldviews value industry and may be inclined to view climate change more sceptically. Thus, for these individuals, the most salient risk in this context is that mitigation efforts might unjustifiably restrict commercial ventures. In contrast, those with stronger egalitarian/communitarian worldviews value equality and tend to support environmental regulation. Thus, for these individuals, the most salient risk is that posed by climate change to the environment and collective wellbeing. Where information appears to be identity-incongruent, biased evaluation of scientific evidence may occur (Kahan and Braman 2006; Kahan et al. 2011). Similar effects have also been observed for other measures of socio-political views: lower acceptance of climate change is also observed among those who endorse free-market economics (e.g. Lewandowsky et al. 2013) and those with conservative political orientations (e.g. McCright et al. 2016; Tranter and Booth 2015). Furthermore, polarisation is not limited to climate change attitudes and beliefs, nor limited to the socio-political right. Public polarisation has also been observed for other topics, such as COVID-19-related public health measures according to cultural tightness (e.g. Sachs et al. 2022) and immigration according to political partisanship (e.g. Saldaña et al. 2021). Polarisation is also sometimes observed among those with liberal political ideologies regarding nuclear energy, genetically modified food safety, and vaccine safety, though findings are mixed, and other research has observed polarisation on these topics among those with conservative political affiliations. This may be due, in part, to cross-cultural variation (e.g. Hamilton 2015; Hornsey et al. 2018; Lobato and Zimmerman 2019). Communicating policy, and in this case, climate policy, in a way that minimises the potential for polarisation, presents an important challenge for policymakers.

Intuitively, increasing citizens’ scientific knowledge should reduce the gap between public and scientific consensus on climate science (Allum et al. 2008). However, for some issues, increased scientific knowledge is associated with increased polarisation along socio-political lines. For example, greater science knowledge is associated with more acceptance of, or concern about, climate change among those with more liberal socio-political ideologies (e.g. Democrat/liberal political affiliation, egalitarian/communitarian worldviews), but less acceptance among those with more conservative socio-political ideologies (e.g. Republican/conservative political affiliation, hierarchical/individualistic worldviews; Ballew et al. 2020; C. Drummond and Fischhoff 2017; Ehret et al. 2017; Hamilton 2011; Hamilton et al. 2015; Kahan et al. 2012). Although scientific knowledge plays an important societal role (Miller 2010), increasing knowledge alone is insufficient for improving climate change reasoning.

Positive attitudes towards science may protect against polarisation for science-related information (Cook and Lewandowsky 2016; A. Drummond et al. 2016; C. Drummond and Fischhoff 2017; Kahan et al. 2017; Motta 2018, 2019). Accordingly, *scientific interest* is positively associated with trust in climate scientists (Motta 2018), and *trust in science* (C. Drummond and Fischhoff 2017) and *science curiosity* (Kahan et al. 2017) are positively associated with science-consistent climate change beliefs. Here, we focus on a particular attitude towards science: *endorsement of scientific inquiry* (ESI; A. Drummond et al. 2016).

### Theoretical rationale

ESI is an attitude whereby scientific research is viewed as a sound and beneficial basis for understanding the world and forming conclusions about matters of societal importance (A. Drummond et al. 2016; Klopfer 1971; Organisation for Economic Cooperation and Development [OECD] 2009, 2017). The concept of ESI is derived from theory regarding attitudes towards science in education (Klopfer 1971), which was further developed in the context of the Programme for International Student Assessment (OECD 2006), and adapted for experimental research in public support for science (A. Drummond et al. 2016). Conceptually, ESI is distinct from other attitudes towards science (Gardner 1975; Klopfer 1971; Osborne et al. 2003). Individuals can endorse scientific inquiry as a valid way to reason without necessarily having an interest in science, participating in scientific pursuits, or having factual scientific knowledge. Furthermore, ESI does not require the ability to perform scientific experiments, simply an appreciation that the scientific method is a valuable way to develop understanding (Klopfer 1971; OECD 2017). Thus, acquiring an attitude whereby one values the processes of science may be relatively simple compared to, for example, acquiring the knowledge and skills to perform the processes of science. A large body of evidence demonstrates that many attitudes are malleable (Eiser 1986; Reid 2006) and can predict behavioural intentions (Ajzen 2001).

Based on these ideas, ESI might provide a useful framework to help us understand why people might support, or not support, certain policies or engage in certain behaviours. Our theoretical rationale is that ESI should be positively associated with evidence-based reasoning and increased alignment between attitudes and scientific evidence. ESI, therefore, seems to be a promising avenue for individuals to more closely consider the scientific evidence for behaviour and policy recommendations. Thus, ESI interventions could potentially be used to shift behavioural intentions across a range of domains. Promisingly—and consistent with these ideas—ESI is malleable, and changes in ESI are positively associated with pro-environmental policy support (A. Drummond et al. 2016). Additionally, a simple intervention can increase ESI and subsequently, climate policy support (A. Drummond et al. 2016). Crucially, these beneficial effects occur independent of cultural worldviews (A. Drummond et al. 2016). Here, we test this theory by examining a particular domain: climate change.

### The current study

Our current research investigated an unanswered question: is higher ESI *also* associated with better discrimination of scientific evidence? In other words, is the scientific strength of evidence more salient for people with higher ESI, such that they adjust their climate policy support according to the quality of evidence, or are they simply more supportive of any climate policy, regardless of evidence quality? If the first proposition is true, individuals with higher ESI may be better at evaluating supporting evidence for policies, suggesting greater engagement of analytical thinking (Evans and Stanovich 2013; Kahneman 2011). This would be particularly beneficial for progress on mitigating climate change if interventions targeting ESI assist people to discriminate between stronger- and weaker-evidenced policies. Alternatively, ESI may be positively associated with support for any policy *presumed* to be evidence-based.

We tested these competing propositions in three studies. Individuals rated their support for climate policies accompanied by stronger or weaker evidence. Better evidence discrimination was operationalised as greater differences in support ratings for stronger- and weaker-evidenced policies. In study 1, we tested whether climate policy evidence discrimination would be moderated by ESI, such that as individuals’ ESI increased, evidence discrimination between strong and weak evidenced policies would improve. Furthermore, we anticipated that these effects would not be influenced by cultural worldviews. We also tested whether a similar pattern would be observed as in other studies between science knowledge and cultural worldviews (e.g. Kahan et al. 2012). Here, we anticipated that those with more hierarchical/individualistic worldviews may be less aligned with scientific evidence in their policy support decisions, compared to those with more egalitarian/communitarian worldviews.

## Method: study 1

### Participants

A target sample size of 500 was determined based on similar studies where significant effects were observed (Cook and Lewandowsky 2016; Cook et al. 2017; Kahan et al. 2010, 2011, 2012, 2017; Kerr and Wilson 2018; van der Linden et al. 2015). Participants were recruited through Prolific Academic (https://www.prolific.co), with data collection occurring between 10 to 13th September, 2019. Participation invitations were sent to 523 individuals registered through Prolific Academic who indicated they were Australian, US, or UK residents; had normal to corrected-to-normal vision; and fluency in English. Participants received GBP1.25 compensation on completion. Twenty participants began the study but did not complete, and as such were excluded from all analyses due to missing data. The final sample was 503 participants (265 female; aged 18 to 69 years, *M* = 34.30, *SD* = 12.17).

### Materials

#### Climate policies

Following a process of review across four raters, 16 policy items were developed to assess participants’ decision-making. Each policy item contained two parts: information, e.g. ‘93% of scientific studies found that replacing high-carbon fuels with biofuels, such as ethanol and biodiesel, in heavy vehicle transport would reduce carbon emissions’; and policy, e.g. ‘Now consider a government policy that would require heavy vehicle transport operators to replace high-carbon fuels with biofuels’.

The information component was manipulated to reflect either weak or strong scientific evidence. Evidence was presented either as the level of consensus among scientists or the number of scientific studies conducted. Evidence was either expressed as a percentage, e.g. ‘23% of scientists agree …’, or a number, e.g.‘15 out of 20 scientific studies found …’, to minimise the impact of differences in mathematical competency. Evidence support values (scientific consensus or studies) for weak policies ranged zero to 40% (or equivalent); and strong policies were in the range of 60 to 100% consensus/support. Standardised wording was used, with items referring to ‘reducing carbon emissions’ or ‘removing carbon dioxide’. Policy wording reflected either an incentive-style, for example, ‘… a policy that would provide incentives to businesses’ or a mandating-style, for example, ‘… a policy that would require businesses to …’.

Policy items were counterbalanced by category (four items for transport; food production; land usage; and carbon capture, utilisation, and storage), and each category contained an item pairing of strength and type of evidence (weak/consensus, strong/consensus, weak/studies, strong/studies). Each of the 16 items had a mandating-style and incentive-style version, and items were randomised, such that the incentive and mandating version appeared equally often across the study.^1^

Policy content was developed based on real information and policy recommendations gathered from sources such as the IPCC (2018), but was designed to reflect less well-known concepts, such as geosequestration, for two reasons. First, to reduce the role of pre-existing knowledge about policies, attitudes towards policies, or other prior exposure. Second, to increase the plausibility of the evidence strength manipulation, as without prior knowledge, individuals would need to reason with the information presented, which would assess the primary hypothesis of the study. The strength of evidence was manipulated according to fictitious rates, and this was another reason to use less well-known concepts so that individuals did not have prior knowledge of this information. References to ‘global warming’ were deliberately avoided to minimise partisan bias (Schuldt et al. 2015).

Support for climate policies was measured on a scale of from 1 — *strongly oppose* to 10 — *strongly support.* The total score ranged from 16 to 160. Pilot testing showed a suitable variation in responses to justify continuation with the final items, with scores ranging from 1 to 10 for both weak (*M* = 4.66, *SD* = 2.76) and strong (*M* = 6.74, *SD* = 2.07) items.

#### ESI scale

The concept of ESI was derived from the Programme for International Student Assessment (PISA) 2006 (OECD 2009), which assessed nearly 400,000 students across 57 countries on scientific literacy, reading literacy, and mathematical literacy (OECD 2009). The ESI scale was adapted for adults by A. Drummond and colleagues (2016), and internal reliability was good, *α* = 0.79. ESI was measured with eight items, for example ‘The development of early warning systems for natural disasters should be based on scientific research’, and agreement with each item was rated from 1 — *strongly disagree* to 7 — *strongly agree*. The total score on this scale ranged from 8 to 56.

#### Scientific literacy scale

The scientific literacy scale consisted of seven items taken from the National Science Board Indicators (National Science Board] 2010) designed to measure basic scientific literacy. This scale has been widely used in previous research to measure general scientific knowledge (Allum et al. 2008) and was used in the study by A. Drummond and colleagues (2016), thus selected for the present study for consistency. Each item required a true or false response, for example ‘Antibiotics kill viruses as well as bacteria’. The total score on this scale ranged from 0 to 7.

#### Cultural cognition worldview (CCW) scales

The CCW scales from previous studies (e.g. Kahan et al. 2010, 2011, 2012) were used to measure the extent to which individuals prescribed to hierarchical/individualist or egalitarian/communitarian worldviews. The scales had high reliability for both individualism-communitarianism, *α* = 0.88, and hierarchism-egalitarianism, *α* = 0.89 (Kahan et al. 2010). The scales consisted of six items for hierarchism-egalitarianism, for example ‘We have gone too far in pushing equal rights in this country’, and six items for individualism-communitarianism, for example, ‘The government interferes far too much in our everyday lives’. Participants indicated their agreement with each statement on a scale from 1 — *strongly disagree* to 6 — *strongly agree.* The total score on each scale ranged from 6 to 36; higher scores indicated greater hierarchical and individualist worldviews, where lower scores indicated greater egalitarian and communitarian worldviews (Kahan et al. 2012).

### Procedure

The study was conducted online using LimeSurvey software. Prior to beginning, participants were requested to remove distractions from their environment, read information carefully, and answer questions honestly. Participants were first randomised into one of two sets of 16 counterbalanced policy items which were presented one-by-one in random order. Policy information was presented first, and participants were asked to check a box to indicate they had read the information. The policy was then displayed, and participants rated their support for the policy on a scale of 1 — *strongly oppose* to 10 —* strongly support.*

After rating all 16 policy items, participants completed the ESI scale (A. Drummond et al. 2016), scientific literacy scale (NSF 2010), and CCW scales (Kahan et al. 2012). Demographic information was collected.

### Data analysis

Statistical analyses were completed using SPSS Statistics 24 (IBM) with the Mediation and Moderation for Repeated Measures (MEMORE) macro (Montoya 2019; Montoya and Hayes 2017; available at https://www.akmontoya.com). MEMORE was developed to accommodate two-condition within-subjects moderation designs (Montoya 2019; Montoya and Hayes 2017) based on the PROCESS model (Hayes 2013). MEMORE calculates moderation based on the methods outlined by Judd et al. (2001), which involves estimating the difference in a repeated-measure outcome variable in each condition and the conditional effects of within-subjects or between-subjects moderators (Montoya 2019). All moderators were mean-centred for interpretability, and 5000 samples were used to generate bootstrapped confidence intervals. Analyses were interpreted as statistically significant at an alpha level of 0.05. Confidence intervals and effect sizes were considered in the interpretation of results, where applicable.

## Results: study 1

**Fig. 1:**
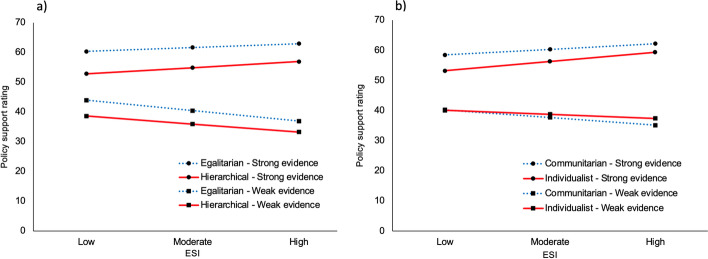
The non-significant interaction between cultural worldviews and ESI on climate policy support. A simple slopes analysis of the non-significant interaction between ESI and worldviews for the **a** hierarchy-egalitarian and **b** individualism-communitarian subscales for weak and strong evidence policy items in study 1. Individualist/hierarchist is defined as 1 SD above the mean on the respective subscale; communitarian/egalitarian is defined as 1 SD below the mean

Study 1 investigated whether higher levels of ESI were associated with better discrimination when evaluating scientific evidence (greater differences in support ratings for weaker-evidenced, compared to stronger-evidenced, policies), and whether this varied depending on cultural worldviews. Descriptive statistics are presented in Supplementary Tables 1 and 2. The within-subjects moderation analysis (Supplementary Fig. 1) revealed that, as ESI increased, participants were more discerning of evidence, giving greater support for climate policies accompanied by stronger evidence, *B* = 0.48 [0.35, 0.60], *t*(501) = 7.19, *p* < 0.001, and less support for climate policies accompanied by weaker evidence, *B* =  − 0.31 [− 0.48, − 0.13], *t*(501) =  − 3.45, *p* < 0.001 (Fig. 1; Supplementary Table 3). These relationships held regardless of individuals’ worldviews.

**Fig. 2:**
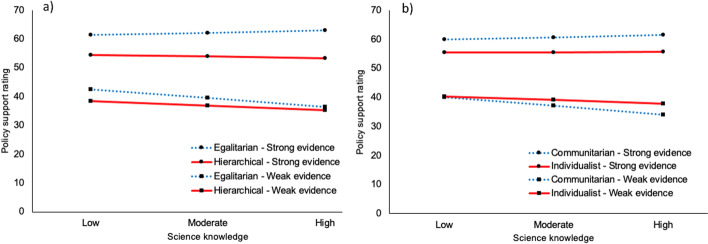
The significant interaction between cultural worldviews and scientific knowledge on climate policy support. A simple slopes analysis of the conditional effects of science knowledge on policy support with weaker and stronger evidence for the **a** hierarchy-egalitarian and **b** individualism-communitarian subscales in study 1. Individualist/hierarchist is defined as 1 SD above the mean on the respective subscale; communitarian/egalitarian is defined as 1 SD below the mean

We then examined the effect of general science knowledge on policy support (Supplementary Fig. 2). In contrast to the results found with ESI as a moderator, the regression analysis identified a significant three-way interaction between evidence strength, science knowledge, and worldviews: unlike for ESI, the moderating effect of science knowledge on the relationship between evidence strength and policy support varied depending on worldviews, *B* = 0.17 [0.02, 0.33], *t*(499) = 2.23, *p* = 0.026. Participants with more egalitarian/communitarian worldviews followed a similar pattern as that observed for ESI, but those with more hierarchical/individualistic worldviews showed a different pattern. For more hierarchical/individualistic participants, increased science knowledge was associated with less, not more, support for stronger-evidenced policies. There was little or no change in support ratings for weaker-evidenced items (Fig. 2). These results were broadly consistent with previous literature, where greater science knowledge has been associated with greater polarisation along socio-political lines (Ballew et al. 2020; C. Drummond and Fischhoff 2017; Ehret et al. 2017; Hamilton 2011; Hamilton et al. 2015; Kahan et al. 2012). To the best of our knowledge, this is the first time that polarisation effects have been shown to differ according to the strength of evidence for a policy: participants with hierarchical/individualistic worldviews reduced their support for policies as the evidence for those policies increased.

## Studies 2 and 3

Study 1 investigated whether there is a positive association between ESI and the ability to discriminate more from less compelling scientific evidence when evaluating climate policy support. Indeed, we found that as ESI increases, so too does discrimination, such that policy evaluation is increasingly in line with scientific evidence with increasing levels of ESI. This was consistent with previous research which suggests that positive attitudes towards science may have a protective effect against polarisation and encourage more evidence-consistent reasoning (Cook and Lewandowsky 2016; A. Drummond et al. 2016; C. Drummond and Fischhoff 2017; Kahan et al. 2017; Motta 2018, 2019).

In studies 2 and 3, we sought to extend on these ideas. Moderated-mediation designs experimentally tested whether a previously successful intervention (A. Drummond et al. 2016) could increase ESI compared to a control group, and whether increases would translate to further improvements in evidence discrimination. We also examined whether cultural worldviews influenced these relationships. We did not have a specific hypothesis in terms of expected effects; however, previous research demonstrates that interventions can fail where the topic of focus (e.g. climate change) is inconsistent with the individual’s ideologies (e.g. Kahan 2013). Given the methodological and conceptual similarity of studies 2 and 3, we present the methods and results from these studies together to assist the reader in identifying the key similarities and difference in findings between studies. Study 3 was a replication of study 2 using alternative recommendations for sample size to clarify the findings of study 2.

## Method: study 2

### Participants

Four hundred and twenty three participants were recruited from Prolific Academic (total eligible = 62,156), with data collection occurring between 29th to 31st July, 2020. Twenty participants were excluded due to non-completion. Three participants failed the attention check; however, these were not excluded as all three failed with the same response (grasshopper) and it was considered that this may have reflected a misunderstanding of the question, rather than lack of attention. The final sample consisted of 403 participants (190 female, 209 male, 3 other; aged 18 to 74 years, *M* = 32.02, *SD* = 11.93). Additional demographic questions regarding education, country of primary residency, and ethnicity were included to provide an indication of representativeness. Participants were again required to have normal to corrected-to-normal vision, and fluency in English. They received GBP1.42 on completion.

### Materials

Study 2 adopted the same materials used in study 1 (climate policies, ESI scale, scientific literacy scale, CCW scale). In addition, for the experimental manipulation, participants either viewed an experimental fact sheet about the benefits of the scientific method, designed to increase ESI, or a control fact sheet about sleep. Each fact sheet was a similar word length (ESI 574 words; sleep 584 words), and each were accompanied by four questions related to the assigned fact sheet, designed to further engage participants in the material. The experimental sheet contained statements such as ‘After Semmelweis introduced a hand washing procedure for doctors after they had worked with a corpse, the mortality rate in the maternity ward drastically decreased. Thus, Semmelweis was able to save lives. Notably, it was by using the scientific method that Semmelweis was able to detect and counteract an invisible cause of infection’. The control sheet contained statements which were also scientific in nature, but not related to the benefits of science for decision-making. For instance, the control sheet stated that ‘If you attach an electroencephalograph to a person’s head, you can record the person’s brainwave activity. An awake and relaxed person generates alpha waves, which are consistent oscillations at about 10 cycles per second. An alert person generates beta waves, which are about twice as fast.’ Thus, both sheets relayed information about scientific facts in a positive way implying their status as factual, but the experimental sheet made specific links to decision-making (see Supplementary Information; A. Drummond et al. 2016).

### Procedure

After accepting the participation invitation, participants were randomised into either an experimental or control condition. Those in the experimental condition read the ESI fact sheet about the scientific method, then were asked to indicate how useful (on a scale from 1 — *not at all useful* to 5 — *extremely useful*) the scientific method was for making decisions about four different tasks. Participants in the control condition read a fact sheet about sleep and were asked how useful four different indicators were to determine whether a person was asleep.

Participants were then randomised into one of two sets of climate policy items as in study 1 and asked to read the policy information and rate their support for 16 policies. Participants then undertook an attention check used in a previous study, which was a single question: ‘Which of these five items is not an animal’, with the options ‘Lion’, ‘Table’, ‘Moose’, ‘Cat’, ‘Grasshopper’ (Lewandowsky et al. 2020). Finally, participants completed the ESI scale, scientific literacy scale, CCW scales, and demographic questions.

## Method: study 3

### Participants

A total of 627 individuals accepted the invitation to participate in the study via Prolific Academic (total eligible = 61,885). Data collection occurred between 12th to 23rd November, 2020. The same inclusion and exclusion criteria applied as in the previous studies, and participants were also excluded if they had previously completed study 1 or study 2. Twenty-seven individuals did not complete and were excluded. All participants passed the attention check. The final sample size was 600 (301 male, 293 female, 5 other, 1 not indicated; aged 18 to 80 years, *M* = 34.67, *SD* = 12.91). Sample size was based on recommendations to detect a small effect (Fritz and MacKinnon 2007; Preacher et al. 2007).

### Materials

The materials and design were identical to study 2, with the exception of two additional questions relating to climate change beliefs, ‘How convinced are you that climate change is happening?’ (1 — *not at all convinced* to 4 — *completely convinced*), and concern, ‘To what extent are you personally worried about climate change?’ (1 — *not at all worried* to 4 — *a great deal worried*). As per the pre-registration, we did not intend to analyse these questions for the current study.

### Procedure

Study 3 followed the same procedure as in Study 2, aside from the additional climate change belief and concern questions, which were asked following completion of the CCW scales and before the demographic questions. The attention check response options were also adjusted given the possibility that this had been misunderstood, with the previous response option ‘Grasshopper’ replaced with ‘Rabbit’.

### Data analysis

Statistical analyses for studies 2 and 3 were completed using SPSS Statistics 28 (IBM) using the PROCESS macro for moderation and mediation (Hayes 2013) and the MEMORE macro for within-subjects moderation (Montoya 2019; Montoya and Hayes 2017).

## Results: studies 2 and 3

Studies 2 and 3 investigated whether an ESI intervention would be associated with improvements in evidence discrimination through increases in ESI. Previous work has shown this intervention increased ESI, where the control did not (A. Drummond et al. 2016). Descriptive statistics are presented in Supplementary Tables 4 and 5.

**Fig. 3:**
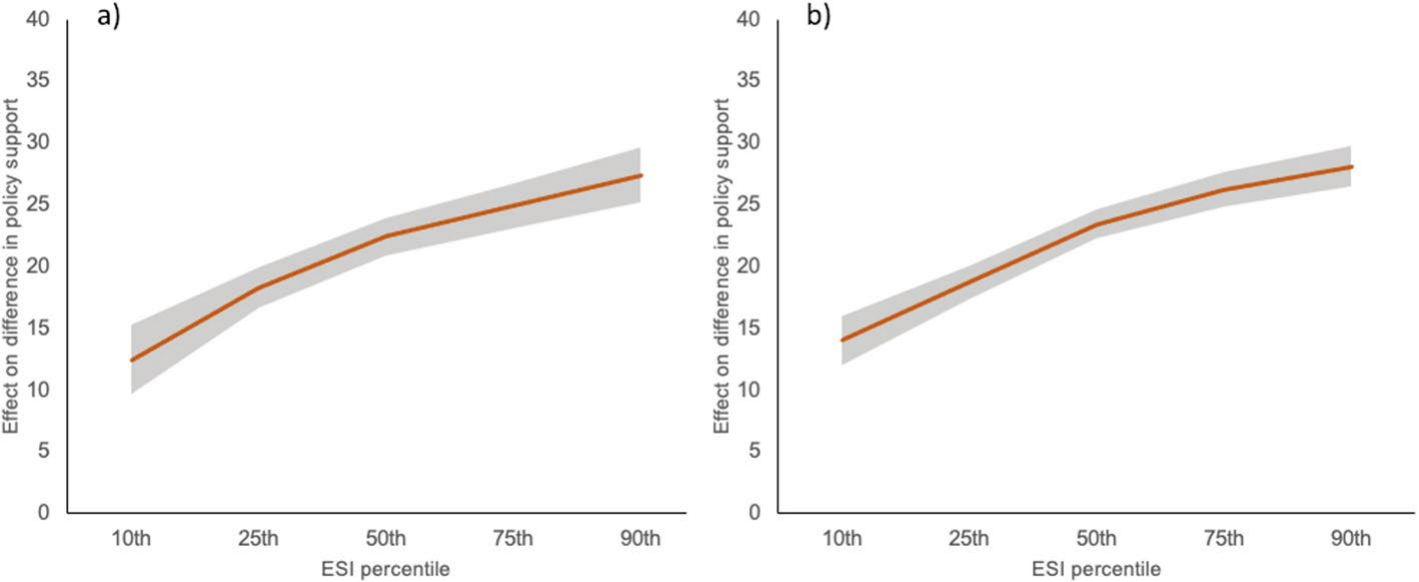
The effect of ESI on climate evidence discrimination. The significant effect of increasing ESI scores on the difference in ratings for policies accompanied by stronger and weaker evidence in **a** study 2 and **b** study 3. The shaded area represents 95% confidence intervals

We hypothesised that the relationship between ESI and improved evaluation of evidence observed in study 1 would replicate to studies 2 and 3. When experimental group was ignored, the within-subjects multiplicative moderation analysis (Supplementary Fig. 1; Montoya and Hayes 2017; Montoya 2019) revealed the same patterns as detected in study 1: as ESI increased, participants gave significantly lower support ratings to weaker-evidenced policies, and significantly higher support ratings to stronger-evidenced policies (Table 1). This relationship was not moderated by worldviews (*ps* > 0.05). Figure 3 shows the significant effect on the difference in ratings for weaker- compared to stronger-evidenced policies for participants according to different percentiles of ESI. This provides strong support for the hypothesis that ESI is positively associated with better evaluation of policy evidence in these contexts, and importantly that ESI appears to function independent of worldview.

**Table 1:** The moderating effect of ESI on climate policy ratings according to evidence strength: higher ESI was associated with less support for weak-evidenced policies, and greater support for strong-evidenced policies

Evidence strength	*B*	SE	*t*	*p*	*95%CI*
Weak evidence					
Study 1	− 0.31	0.09	− 3.45	< 0.001	[− 0.48, − 0.13]
Study 2	− 0.34	0.10	− 3.33	< 0.001	[− 0.54, − 0.14]
Study 3	− 0.28	0.09	− 3.01	0.003	[− 0.46, − 0.10]
Strong evidence					
Study 1	0.48	0.07	7.19	< 0.001	[0.35, 0.60]
Study 2	0.49	0.08	6.07	< 0.001	[0.33, 0.65]
Study 3	0.66	0.07	9.35	< 0.001	[0.52, 0.80]

*Note. CI*, confidence interval

Next, we examined the effects of the ESI intervention and whether these effects were moderated by worldviews. Moderated mediation analyses (PROCESS Model 15; Supplementary Fig. 3; Hayes 2013) revealed non-significant effects for individualist/communitarians (study 2, 95% CI [− 0.07, 0.09]; study 3, 95% CI [− 0.03, 0.04]) and hierarchical/egalitarians (study 2, 95% CI [− 0.06, 0.05]; study 3, 95% CI [− 0.01, 0.02]). Thus, the effects of experimental group on policy support, and ESI on policy support, were not moderated by worldviews. This is consistent with the effects of ESI on policy support being independent of worldviews (A. Drummond et al. 2016).

A second set of mediation analyses omitting the moderator (PROCESS Model 4; Hayes 2013) revealed a significant effect of ESI on difference scores in both study 2, *B* = 0.81 [0.58, 1.03], *t*(399) = 6.99, *p* < 0.001, and study 3, *B* = 0.93 [0.75, 1.12], *t*(597) = 9.93, *p* < 0.001, such that as ESI increased, the difference in support for policies with stronger and weaker evidence increased. There was also a significant effect of experimental group on difference scores in both study 2, *B* = 3.55 [0.66, 6.44], *t*(399) = 2.42, *p* = 0.016, and study 3, *B* = 4.69 [2.46, 6.93], *t*(597) = 4.12, *p* < 0.001, such that differences in support for policies with stronger, compared to weaker, evidence were significantly greater for participants in the ESI intervention group compared to the control group (see Supplementary Tables 6 and 7).

However, in terms of whether the intervention actually increased ESI and this in turn led to differences in policy evaluation, the effect of experimental group on ESI was non-significant in both instances (study 2, *B* = 1.16 [− 0.09, 2.41], *t*(400) = 1.82, *p* = 0.069; study 3, *B* = 0.20 [− 0.78, 1.17], *t*(598) = 0.40, *p* = 0.691), and the crucial indirect effect of experimental group on policy support through ESI was also non-significant for both studies (study 2, *B* = 0.93 [− 0.09, 2.03]; study 3, *B* = 0.18 [− 0.72, 1.09]). Thus, the mediation model we hypothesised was not supported. However, given difference scores for policy support were significantly higher in both studies for the ESI intervention group (study 2, *M* = 23.42, *SD* = 15.94, *d* = 0.29; study 3, *M* = 24.49, *SD* = 15.37, *d* = 0.32) compared to the control group (study 2, *M* = 18.94, *SD* = 15.10; study 3, *M* = 19.62, *SD* = 14.69), there is evidence that the intervention ultimately achieved the desired effect, but the mechanisms may be more complex than first hypothesised.

**Fig. 4:**
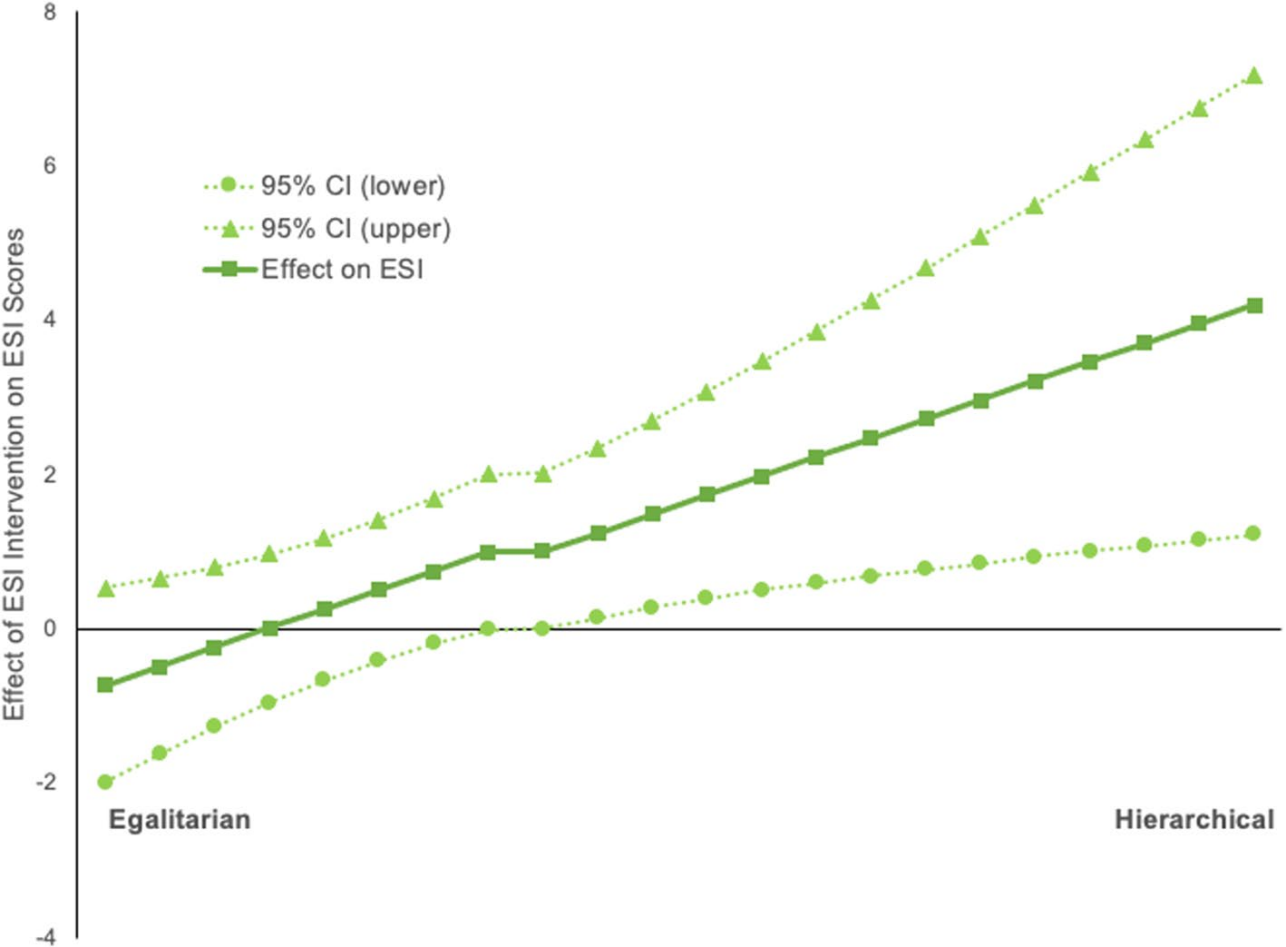
The effect of an ESI intervention on ESI scores according to cultural worldviews. The conditional effect of cultural worldviews (egalitarian/hierarchical) on changes to ESI scores following an intervention (study 3). The dotted lines represent 95% confidence intervals

To further understand the mechanisms underlying the differences between treatment groups, we conducted additional exploratory analyses. When we investigated whether worldviews moderated the effect of the treatment group on ESI (Hayes 2013; PROCESS Model 8, Supplementary Fig. 4), we found that they did in study 3. The conditional effect of worldviews on ESI was non-significant for participants who were more egalitarian (lower scores on the hierarchism-egalitarianism scale). However, as scores increased, indicating stronger hierarchical worldviews, the effect of the intervention on ESI became significant and strengthened (Fig. 4). The *R*^2^ value indicated that this was a moderate effect (*R*^*2*^ = 0.13; Cohen 1988). We also observed a significant moderated mediation for hierarchical-egalitarian worldviews for study 3, *B* = 0.14 [0.01, 0.27], which indicates that the indirect effect—the effect of the intervention on difference scores through ESI—differed depending on worldviews.

Given that differences in the strength of climate change polarisation have been observed cross-culturally, and stronger polarisation has been observed in the USA in particular (Hornsey 2021), we considered whether the effect of the ESI intervention on ESI might be influenced by country of residency.^2^ For the UK participants in study 3 (*N* = 245), consistent with the overall results, increases in ESI were associated with increases in difference scores, *B* = 0.54 [0.23, 0.84], *t*(242) = 3.47, *p* < 0.001. The intervention also did not significantly influence ESI,* B* = 0.05 [− 1.37, 1.47], *t*(243) = 0.07, *p* = 0.942, and the indirect effect was not significant [− 0.06, 0.06]. Similar to the overall results, although the ESI intervention did not shift ESI scores in the UK sample, the direct effect of the intervention on difference scores was significant, *B* = 5.69 [2.29, 9.11], *t*(242) = 3.29, *p* = 0.001. Moreover, the lack of a significant interaction between experimental group and hierarchical-egalitarian worldviews, *B* = 0.19 [− 0.29, 0.66], *t*(240) = 0.77, *p* = 0.441, indicated that the effect of the intervention on difference scores did not significantly differ between participants with more egalitarian, *B* = 4.49 [− 0.41, 9.40], *t*(240) = 1.81, *p* = 0.072, moderate, *B* = 5.60 [2.11, 9.09], *t*(240) = 3.17, *p* = 0.002, and hierarchical worldviews, *B* = 7.26 [2.33, 12.19], *t*(240) = 2.90, *p* = 0.004. Thus, although the intervention did not influence ESI scores, it did influence policy evidence discernment, and this effect did not differ with varying worldviews.

Interestingly, a somewhat different pattern of results emerged for the US sample in study 3 (*N* = 330). There were consistencies in terms of the overall effect of the ESI intervention on difference scores, *B* = 3.87 [0.76, 6.97], *t*(327) = 2.45, *p* = 0.015, and the relationship between ESI and difference scores, *B* = 1.14 [0.90, 1.38], *t*(327) = 9.36, *p* < 0.001. However, the effect of the ESI intervention on ESI was different to the UK sample. There was a significant interaction between experimental group and hierarchical-egalitarian worldviews in terms of their effects on ESI, *B* = 0.30 [0.14, 0.47], *t*(326) = 3.69, *p* < 0.001, such that the intervention had a positive effect on ESI among those with stronger hierarchical worldviews, *B* = 2.96 [1.15, 4.76], *t*(326) = 3.21, *p* = 0.001, and this effect strengthened as hierarchical worldviews increased. Furthermore, a significant moderated mediation was observed, *B* = 0.32 [0.10, 0.55], suggesting that for participants with more hierarchical worldviews, the ESI intervention improved evidence discernment via differences in ESI.

When we conducted a similar analysis using individualist-communitarian worldviews as a moderating variable, a significant interaction was also observed between experimental group and individualist-communitarian worldviews on ESI, *B* = 0.22 [0.00, 0.44], *t*(326) = 1.98, *p* < 0.048, such that a significant effect of the ESI intervention on ESI was observed among participants with relatively high scores on the individualist-communitarian scale (i.e. more individualistic), but this effect was less pronounced compared to hierarchical-egalitarian worldviews, and the moderated mediation was not significant, *B* = 0.25 [− 0.00, 0.51]. Nevertheless, the overall pattern of results from these two analyses suggests that the ESI intervention may have had a meaningful effect on ESI among US participants with more hierarchical/individualistic worldviews, but not those with more egalitarian/communitarian worldviews. This is a promising finding, given that problematic polarisation tends to occur among those with more hierarchical/individualistic worldviews and conservative political affiliations with climate change information (Ballew et al. 2020; C. Drummond and Fischhoff 2017; Ehret et al. 2017; Hamilton 2011; Hamilton et al. 2015; Hornsey 2021; Kahan et al. 2012), and this tends to be more pronounced in the US (Hornsey 2021). However, as this pattern was not consistently observed for studies 2 and 3, this interpretation is only speculative at this stage.

In summary, we found a consistent, positive relationship between ESI and discrimination of evidence, independent of worldviews: higher ESI was associated with policy ratings that better reflected strength of evidence. Furthermore, our intervention also improved discrimination of scientific evidence. However, contrary to expectations, the results of our intervention were not consistent according to cultural worldviews or country of residency. Exploratory analyses suggested that, for more hierarchical/individualistic participants in the USA, our intervention increased ESI, and this increase translated to improved policy evidence discrimination. In contrast, for more egalitarian/communitarian participants, the intervention increased evidence discrimination without affecting ESI. This effect was not observed for UK participants, such that the ESI intervention appeared to have a more direct effect on policy ratings and was associated with better discernment for policies accompanied by stronger or weaker evidence. Thus, a simple intervention can improve ESI and discrimination between strong and weak scientific evidence, but the mechanisms underpinning this effect appear to differ with cultural worldviews and country of residency.

## Discussion

This research addressed two important questions. First, do individuals who endorse scientific inquiry as a way of understanding the world make ‘better’ decisions when confronted with scientific evidence? Second, does increasing endorsement of scientific inquiry lead to improved discrimination of scientific evidence? Across all three studies, we consistently found significant positive relationships between ESI and discrimination of scientific evidence related to climate policies: as participants’ ESI increased, the difference in support for climate policies with stronger (versus weaker) evidence also increased. Importantly, this positive relationship occurred for hierarchical/individualist and egalitarian/communitarian participants. Thus, individuals with higher ESI are better at discerning scientific evidence regardless of worldviews. In contrast, those with higher science knowledge appeared to prioritise worldviews over the strength of evidence when rating support for policies (note that we report these results for study 1; results for studies 2 and 3 are available on request). These results are consistent with the idea that individuals who are higher in ESI place greater weight on the strength of scientific evidence. These novel findings extend on previous research and demonstrate that higher levels of ESI may not only help to override the influence of worldviews and garner greater support for climate policies (A. Drummond et al. 2016), but may also be associated with improved evaluation of climate change information in a way that is more consistent with scientific evidence. This is encouraging for future efforts to increase public support for evidence-based climate policies.

Promisingly, we also found an effect of our intervention on policy support, with greater differences in policy support ratings for participants who received the ESI intervention. However, the mechanisms underpinning the effect of the intervention were more complex than hypothesised, and differed between hierarchical/individualistic and egalitarian/communitarian participants. In study 3, for hierarchical/individualistic participants, the intervention operated as expected: ESI increased which, in turn, increased policy discernment. However, for egalitarian/communitarian participants, the intervention did not significantly increase ESI, but it did improve evidence discrimination. This may have been because egalitarian/communitarian participants were already relatively high in ESI. A median split showed that egalitarian/communitarian participants were significantly higher in ESI (egalitarians: *M* = 50.78, *SD* = 4.71; communitarians: *M* = 49.88, *SD* = 5.25) compared to hierarchical/individualistic participants (hierarchical: *M* = 46.63, *SD* = 6.42; individualists: *M* = 47.20, *SD* = 6.50). Thus, for egalitarian/communitarian participants, it is plausible that the ESI intervention increased the salience of scientific evidence, or the importance of scientific evidence when evaluating policies, even in the absence of changes in ESI. However, on closer inspection, it also appeared that country of residency was important: improved policy discernment through increases in ESI was observed for those in the USA, but not in the UK. Therefore, a better explanation may be that the relatively stronger public consensus on climate change in the UK might be creating some ceiling effects; thus, the effect was observed for the US participants, but not the UK participants. Alternatively, this may highlight potential cross-cultural differences with interventions designed to shift attitudes towards science. Though we are unable to conclude whether changes in ESI or evidence discrimination can be sustained over time, even if the ESI intervention is serving as a temporary prompt to engage in an evidence-based decision, this is promising. Our findings for hierarchical/individualistic participants were important, as the literature demonstrates that those with more conservative socio-political views tend to become polarised in the opposite direction of climate change evidence (Ballew et al. 2020; Druckman and McGrath 2019; C. Drummond and Fischhoff 2017; Ehret et al. 2017; Hamilton 2011; Hamilton et al. 2015; Hornsey 2021; Kahan et al. 2012). This presents a useful direction for future research.

The current study investigated the association between ESI and deliberation with climate policy evidence using the cultural cognition worldview scales (Kahan et al. 2010, 2011). While this is a widely used and valid measure of worldviews, and there is evidence that cultural worldviews are associated with public acceptance of climate change, this association may be more pronounced in Western democracies, particularly those with greater reliance on fossil fuels (see Hornsey 2021). This may present a limitation of the current research; thus, investigating these ideas with other measures of worldviews (e.g. free-market endorsement), political orientations, or other cross-cultural differences may provide a more complete view of these interactions.

In the present work, participants were asked to rate their support for hypothetical climate policies accompanied by hypothetical information (based on real information to an extent). Although these results may reflect an approximation of public decision-making (i.e. within a closed system), further work is needed to test the extent to which responses apply to evaluations within open-system contexts, for example, where complex and competing information is available via media sources. Furthermore, polarisation is not limited to climate change, and there are a number of areas where public opinion is divided, which can vary cross-culturally, e.g. COVID-19-related public health measures (Sachs et al. 2022), immigration (Saldaña et al. 2021), nuclear energy, genetically modified food safety, and vaccine safety (e.g. Hamilton 2015; Hornsey et al. 2018; Lobato and Zimmerman 2019). Some cross-cultural constructs have relatively consistent associations across a range of decision-making contexts (e.g. cultural cognition, Kahan and Braman 2006), while the effects of other constructs vary between decision-making contexts (e.g. cultural tightness, Drummond et al. 2021). Examining the relationship between ESI and evidence-based decision-making in other contexts, as well as the efficacy of the ESI intervention, presents a valuable opportunity for future research.

It should also be noted that most of the participants were at the higher end of the ESI scale, with the mean ESI score ranging from around 47 to 48 out of a maximum of 56 for the three studies. It is possible that recruiting participants through Prolific Academic may have attracted individuals with a tendency to have stronger pro-science attitudes. However, this research participation platform is a valid and reliable method of data collection (Peer et al. 2017), and if pro-science attitudes were relatively high in this sample, this would simply serve as a more conservative test of our intervention. Crucially, however, even a small amount of variability in the scale was meaningful: even relatively minor differences in ESI were associated with improved evidence evaluation and policy support that was better aligned with the strength of scientific evidence. Nevertheless, testing the efficacy of the ESI intervention with populations where pre-existing attitudes towards science may be relatively low presents an interesting question for future research. Furthermore, improving the sensitivity of the scale may be warranted, and efforts to increase the strength of effects remain a worthy goal.

In summary, we revealed a novel finding that individuals who place higher value on the scientific method as a basis for understanding (i.e. ESI) are able to make decisions that better align with the strength of evidence associated with climate policies. Promisingly, ESI appears to override the effect of worldviews when evaluating scientific evidence, whereas basic science knowledge did not. This effect was observed across all three studies. Additionally, we demonstrated that a simple intervention successfully increased ESI among US participants with more hierarchical/individualistic worldviews and this translated to improved discrimination of scientific evidence. Overall, these results indicate that directing resources towards practical strategies to increase ESI may be an effective way to elicit climate policy support (A. Drummond et al. 2016). That such a simple intervention improved discernment between strong- and weak-evidenced policies is promising, and this presents opportunities for more comprehensive ESI interventions to be developed. If resources can be directed towards increasing ESI, this may not only increase but also expedite, public support for quality pro-environmental policies, and assist individuals to manage climate change misinformation (A. Drummond et al. 2016).

Although our focus in this research was climate change specifically, we believe that ESI will be applicable across a variety of domains, such as policies to counteract pandemics (e.g. COVID-19), election misinformation and conspiracy theories, and public health interventions more broadly. Because findings observed for attitudes towards climate change do not always translate to other domains (A. Drummond et al. 2021; Lewandowsky et al. 2013; Rutjens et al. 2018), future empirical work will be required to test the extent to which ESI is a useful construct in these domains. However, pending such work, we suggest that ESI might be a promising vehicle for boosting support for well-evidenced policies in other domains.

## Supplementary Information

Below is the link to the electronic supplementary material.

Supplementary file1 (PDF 172 KB)

Supplementary file2 (DOCX 21 KB)

## Data Availability

Study 1 was not preregistered; Study 2 (https://osf.io/j32zx) and Study 3 (https://osf.io/wpn49) were both preregistered. The deidentified data for Studies 1, 2, and 3, along with data analysis scripts and materials, are 10.1007/s10584-023-03535-y available to qualified researchers on request.

## Footnotes

1 We considered that the style of wording may influence individuals’ evaluations of policy support, according to cultural worldviews. This was analysed for study 1, but we did not find a significant effect of policy wording, or any significant interactions between policy wording and cultural worldviews. This indicated that policy wording did not elicit a meaningful effect, therefore this was not analysed for studies 2 and 3.

2 We thank an anonymous reviewer for suggesting this. As the majority of the sample consisted of participants residing in the UK and USA, we did not separately analyse the remaining participants (*N* = 25) due to insufficient power.
