# Supplementary material for: Endorsement of scientific inquiry promotes better evaluation of climate policy evidence
Source: Clim Change. 2023 May 19;176(6):69. doi: 10.1007/s10584-023-03535-y (PMC10197046; doi:10.1007/s10584-023-03535-y)

Supplementary tables

Supplementary Table 1

Study 1: Means, Standard Deviations, and Range for Policy Support Ratings

			Score range	
Evidence strength	*M*	*SD*	Minimum	Maximum
Weak evidence	38.03	13.49	8	80
Strong evidence	58.11	10.39	13	80

Supplementary Table 2

Study 1: Summary of Means, Standard Deviations, and Range for Scores on the ESI Scale, Scientific Literacy Scale, and Cultural Cognition Worldview Scales

			Score range	
Scale	*M*	*SD*	Minimum	Maximum
ESI	47.16	6.70	23	56
Scientific Literacy	5.71	1.19	2	7
Cultural Cognition Worldview Scale				
Individualism-communitarianism	21.13	5.42	6	36
Hierarchism-egalitarianism	14.32	7.20	6	36

Supplementary Table 3

Study 1: Simple Slopes Analysis of the Effect of Evidence Strength on Support at Levels of ESI

					95% CI	
ESI	Effect	*SE*	*t*	*p*	*LL*	*UL*
-9.16	-12.93	1.07	-12.10	<.001	-15.03	-10.83
-4.16	-16.84	.74	-22.67	<.001	-18.30	-15.38
.84	-20.74	.64	-32.62	<.001	-21.99	-19.49
5.84	-24.64	.84	-29.44	<.001	-26.29	-23.00
7.84	-26.21	.97	-26.98	<.001	-28.11	-24.30

Note. 95% CI = bootstrapped confidence interval; *LL* = lower limit, *UL* = upper limit. Greater negative values for effect indicate greater difference in support between Weak and Strong items. Values of ESI represent values in the 10th, 25th, 50th, 75th, and 90th percentile.

Supplementary Table 4

Study 2: Summary of Means, Standard Deviations, and Range for Scores on the ESI Scale, Scientific Literacy Scale, and Cultural Cognition Worldview Scales by Treatment Group

Scale		*M*	*SD*	Score range	
				Minimum	Maximum
ESI					
	ESI group	48.00	6.20	29	56
	Control group	46.84	6.51	31	56
	Total	**47.40**	**6.38**	**29**	**56**
Scientific Literacy					
	ESI group	5.72	1.30	2	7
	Control group	5.75	1.23	1	7
	Total	**5.74**	**1.26**	**1**	**7**
CCWS – Individualism-communitarianism					
	ESI group	19.64	5.40	6	34
	Control group	20.21	4.91	8	34
	Total	**19.93**	**5.15**	**6**	**34**
CCWS – Hierarchism-egalitarianism					
	ESI group	13.11	7.40	6	36
	Control group	14.07	7.00	6	35
	Total	**13.61**	**7.20**	**6**	**36**

Supplementary Table 5

Study 2: Summary of Means, Standard Deviations, and Range for Scores on the ESI Scale, Scientific Literacy Scale, and Cultural Cognition Worldview Scales by Treatment Group for Study Three

Scale		*M*	*SD*	Score range	
				Minimum	Maximum
ESI					
	ESI group	48.61	5.74	27	56
	Control group	48.42	6.37	24	56
	Total	**48.51**	**6.06**	**24**	**56**
Scientific Literacy					
	ESI group	5.85	1.15	2	7
	Control group	5.92	1.15	2	7
	Total	**5.89**	**1.15**	**2**	**7**
CCWS – Individualism-communitarianism					
	ESI group	19.85	5.88	6	36
	Control group	19.83	5.56	6	36
	Total	**19.84**	**5.72**	**6**	**36**
CCWS – Hierarchism-egalitarianism					
	ESI group	13.45	7.59	6	36
	Control group	12.65	7.14	6	36
	Total	**13.04**	**7.37**	**6**	**36**

Supplementary Table 6

Study 2: Means, Standard Deviations, and Range for Policy Support Ratings for Weak and Strong-evidenced Policies by Experimental Group, Control Group, and Total Sample

Evidence strength	*n*	*M*	*SD*	Score range	
				Minimum	Maximum
Weak evidence					
ESI group	193	36.74	13.61	14	80
Control group	209	38.68	12.94	12	74
Total	**402**	**37.75**	**13.28**	**12**	**80**
Strong evidence					
ESI group	193	60.17	10.05	22	80
Control group	209	57.62	11.21	14	80
Total	**402**	**58.84**	**10.73**	**14**	**80**

Supplementary Table 7

Study 3: Means, Standard Deviations, and Range for Policy Support Ratings for Weak and Strong-evidenced Policies by Experimental Group, Control Group, and Total Sample

Evidence strength	*n*	*M*	*SD*	Score range	
				Minimum	Maximum
Weak evidence					
ESI group	294	34.39	13.77	8	70
Control group	306	38.58	13.74	8	80
Total	**600**	**36.53**	**13.90**	**8**	**80**
Strong evidence					
ESI group	294	58.88	10.26	8	80
Control group	306	58.19	12.03	8	80
Total	**600**	**58.53**	**11.19**	**8**	**80**

Supplementary figures

Supplementary Figure 1

A Multiplicative Moderation Model for ESI and Worldviews. *Examining whether ESI and worldviews moderate the relationship between support for policies and the strength of evidence*

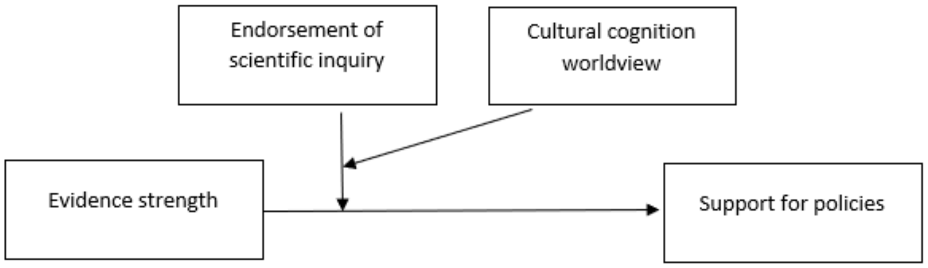

Supplementary Figure 2

A Multiplicative Moderation Model for Scientific Knowledge and Worldviews. *Examining whether SciLit and worldviews moderate the relationship between support for policies and the strength of evidence*

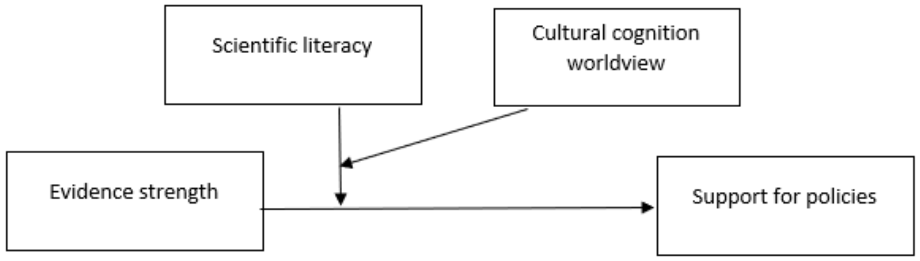

Supplementary Figure 3

A Moderated Mediation Model (PROCESS Model 15) for ESI and Worldviews. *Examining whether worldviews moderate the mediating relationship of experimental group on support for policies through ESI*

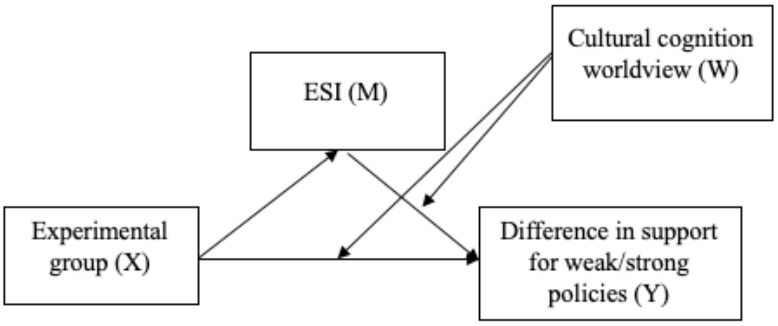

Supplementary Figure 4

A Moderated Mediation Model (PROCESS Model 8) for ESI and Worldviews. *Examining whether worldviews moderate the mediating relationship of experimental group on support for policies through ESI*

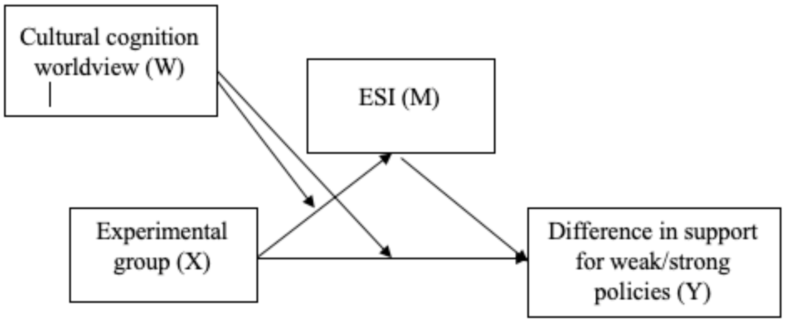

Supplement: Supplementary file 1 — Supplementary file1 (PDF 172 KB) [file 10584_2023_3535_MOESM1_ESM.pdf]
